# Imaging Alzheimer's disease pathophysiology with PET

**DOI:** 10.1590/S1980-5764-2016DN1002003

**Published:** 2016

**Authors:** Lucas Porcello Schilling, Eduardo R. Zimmer, Monica Shin, Antoine Leuzy, Tharick A. Pascoal, Andréa L. Benedet, Wyllians Vendramini Borelli, André Palmini, Serge Gauthier, Pedro Rosa-Neto

**Affiliations:** 1 Translational Neuroimaging Laboratory (TNL), McGill Center for Studies in Aging (MCSA), Douglas Mental Health Research Institute, Montreal, Canada.; 2 Alzheimer's Disease Research Unit, MCSA, Douglas Mental Health Research Institute, Montreal, Canada.; 3 Brain Institute of Rio Grande do Sul, Pontifical Catholic University of Rio Grande do Sul (PUCRS), Porto Alegre RS, Brazil.; 4 Department of Biochemistry, Federal University of Rio Grande do Sul (UFRGS), Porto Alegre RS, Brazil.; 5 Department NVS, Centre for Alzheimer Research, Division of Translational Alzheimer Neurobiology, Karolinska Institutet, Stockholm, Sweden.

**Keywords:** Alzheimer's disease, positron emission tomography, amyloid imaging, neuroinflammation, neurodegeneration, tau

## Abstract

Alzheimer's disease (AD) has been reconceptualised as a dynamic pathophysiological process characterized by preclinical, mild cognitive impairment (MCI), and dementia stages. Positron emission tomography (PET) associated with various molecular imaging agents reveals numerous aspects of dementia pathophysiology, such as brain amyloidosis, tau accumulation, neuroreceptor changes, metabolism abnormalities and neuroinflammation in dementia patients. In the context of a growing shift toward presymptomatic early diagnosis and disease-modifying interventions, PET molecular imaging agents provide an unprecedented means of quantifying the AD pathophysiological process, monitoring disease progression, ascertaining whether therapies engage their respective brain molecular targets, as well as quantifying pharmacological responses. In the present study, we highlight the most important contributions of PET in describing brain molecular abnormalities in AD.

## RESUMO

A doença de Alzheimer tem sido reconceitualizada como um processo patofisiológico dinâmico caracterizado pelos estágios pré-clínico, comprometimento cognitivo leve e demência. A tomografia por emissão de pósitrons associada a vários agentes de imagem molecular revela numerosos aspectos da patofisiologia da demência tais como amiloidose cerebral, acúmulo de tau, mudanças em neurorreceptores, anormalidades de metabolismo e neuroinflamação nestes pacientes. No contexto de um crescimento em direção ao diagnóstico precoce pré-sintomático e intervenções modificadores da doença, a imagem de PET com agentes moleculares fornece meio para quantificar o processo patofisiológico da DA sem precedentes, monitorizar a progressão da doença, bem como quantificar resposta farmacológica. Aqui, nós realçamos as mais importantes contribuições do PET na descrição de anormalidades moleculares cerebrais na DA.

## INTRODUCTION

Alzheimer's disease (AD) was first described in 1906, when the German psychiatrist Alois Alzheimer reported the clinical and pathological features from a patient called Auguste Deter. At age 51, she became afflicted by an obscure progressive neuropsychiatric condition characterized by severe cognitive decline associated with behavioral symptoms. Five years after admission, she became mute and confined to her bed. Similarly to many patients, her death came as a consequence of septicemia. At the necropsy, the brain histopathological examination revealed the presence of amyloid plaques and neurofibrillary tangles, both of which later became known as the neuropathological hallmarks of AD. The term "Alzheimer's disease" was introduced by the psychiatrist Emil Kraepelin in 1910, in his Handbook of Psychiatry.^1^ ^,^ ^2^ Initially considered a rare disease, AD became recognized as a frequent condition in aging individuals as well as the leading cause of dementia.^3^

The neuropathological features of AD constitute the extracellular deposition of amyloid-b (Ab) aggregates (senile plaques), intracellular inclusions of hyperphosphorylated tau aggregates (neurofibrillary tangles; NFTs), brain atrophy and cell depletion.^4^ These features silently accumulate and propagate across brain regions for many years, leading to subsequent clinical and functional decline. At the stage of clinical symptoms of dementia, these pathophysiological processes have already significantly compromised a large proportion of brain circuits involved in cognition. In its typical presentation, AD is characterized by progressive cognitive impairment initially confined to the episodic memory system. 

The research criteria for the diagnosis of AD were first defined in 1984 by a working group jointly established by the National Institute of Neurological and Communicative Disorders and Stroke (NINCDS) and the Alzheimer's Disease and Related Disorders Association (ADRDA).^5^ The NINCDS-ADRDA criteria assumed a static association between the pathological and clinical characteristics of AD.^6^ These criteria were useful and widely adopted, remaining in use for over 25 years. During the last decade, important advances in genetics, biochemistry, clinical, and pathological characterization of dementias have taken place, including the development of *in vivo* biomarkers of AD pathophysiology, leading to changes in the criteria for AD.^6^ An International Working Group (IWG) initiated these revisions,^7^ ^,^ ^8^ followed by the National Institute on Aging and Alzheimer's Association workgroup (NIA-AA), leading to new diagnostic criteria including predementia stages of AD. In particular, the NIA-AA research criteria encompasses asymptomatic "preclinical" AD,^9^ mild cognitive impairment (MCI) due to AD,^10^ and AD dementia stages.^11^ This disease framework incorporates important advances such as the notion of clinical and pathophysiological progression, the genetic form of AD, as well as atypical presentations of AD. Importantly, AD pathophysiology now assumes a progressive cascade of events associated with Ab toxicity, which triggers a series of downstream biochemical cascades including tau hyperphosphorylation, synaptic depletion,^12^ ^,^ ^13^ neuroinflammation,^14^ and abnormal neurotransmission.^15^

Positron emission tomography (PET) is a non-invasive method capable of quantifying biological processes based on the dynamic distribution of radiotracers injected during the scanning session. There are numerous PET molecular imaging agents, each of which is specifically designed to quantify a single molecular target. They allow for the characterization of abnormal protein aggregation (fibrillary Ab or hyperphosphorylated tau deposits), metabolic abnormalities (glucose metabolism and cerebral blood flow), and neuroinflammation (astrocytosis, microgliosis and phospholipase activity). This review article focuses on PET molecular imaging in AD, reviewing the role of imaging biomarkers in the diagnosis and monitoring of key pathophysiological events of AD, which include Ab and tau deposition, neurodegeneration, and neuroinflammation.

## PET BIOMARKERS FOR AMYLOID DEPOSITION

Though the pathogenesis of AD remains unclear, the hallmark of this neurodegenerative disease is the deposition of Ab plaques, together with other features, such as the presence of NFTs. Ab deposits are known to progressively accumulate in certain brain regions over the course of the disease, beginning long before the clinical onset. The canonical PET molecular agent capable of detecting fibrillary Ab *in vivo* is the carbon-11 labelled thioflavin T derivative 2-(4'-methylaminophenyl)-6-hydroxybenzothiazo le, also known as [^11^C]Pittsburgh Compound-B ([^11^C]PiB). The most widely studied amyloid PET tracer, [^11^C]PiB is considered the benchmark for PET-amyloid imaging.^16^ ^-^ ^18^ The short half-life of carbon-11 (20 minutes), however, limits its use to centers possessing an on-site cyclotron and specialized radiochemistry. The new generation of amyloid ligands, labeled with fluorine-18, have a longer half-life of approximately 110 minutes. Due to these differences in half-life, these fluorine-18 labeled compounds can be regularly produced at a cyclotron site and distributed to other facilities (Table 1).^19^ ^,^ ^20^

**Table 1:** Summary of amyloid imaging agents currently available for quantifying brain amyloid.

	[**^11^**C]PIB	[**^18^**F]Flutemetamol	[^18^F]Florbetapir	[**^18^**F]Florbetaben	[**^18^**F]NAV4694
	Research Use	Vizamyl^(r)^	Amivid^(r)^	Neuraceq^(r)^	Phase 3
Alternative name	-	GE-067	-	BAY-94-9172, AV-1	[^18^F]AZD4694
Parent molecule	Benzothiazole	Benzothiazole	Styrylpyridine	Stilbene	Benzothiazole
Amyloid affinity (Ki, nM)	0.9	0.7	2.2.	2.4	0.7
Plasma metabolites	Polar	Polar	Polar and non-polar	Polar and non-polar	Polar
Typical injected dose (MBq)	250-450	250-450	300	300	300
Typical imaging time (min)	40-90	90-110	50-70	90-130	50-60

Previous studies indicate that patients with MCI present 20-30% higher prevalence of amyloid positivity when compared to controls, which suggests that both amnestic and nonamnestic MCI are associated with an increased risk for AD. This association is much more relevant in the amnestic MCI subtype, but it is important to highlight that a large number of MCI patients are amyloid negative, supporting the theory that MCI is not always due to amyloid-related AD pathology.^21^ ^-^ ^25^ A positive amyloid-PET scan increases the probability of conversion to AD;^21^ ^,^ ^26^ ^,^ ^27^ however, the interval over which MCI amyloid positive patients may convert to AD dementia is variable, ranging from 1 to 5 years.^21^ ^,^ ^26^ It is important to keep in mind the present limited clinical utility of detection of AD pathophysiology in MCI patients, given the lack of efficacious therapy as well as the ethical limitations when proposing amyloid imaging in patients meeting clinical criteria for MCI. On the other hand, positive amyloid-PET scans in MCI patients provides confirmation that this clinical situation occurs due to AD pathophysiology, supporting the idea of investing in non-pharmacological interventions, such as cognitive enrichment and diet and lifestyle changes. By contrast, a negative amyloid-PET scan in patients presenting cognitive impairment suggests other non-AD pathologies, such as frontotemporal lobar degeneration, hippocampal sclerosis, or argyrophilic grain disease. Although Parkinson's disease (PD) and dementia with Lewy bodies (DLB) are neurodegenerative disorders associated with brain deposition of fibrillary aggregates of a-synuclein protein, individuals with these synucleinopathies and cognitive decline have shown variability with regard to the presence of an abnormal amyloid-PET scan.^28^ ^,^ ^29^ Individuals presenting with Parkinson's disease (PD) dementia have shown lower brain amyloid burden than AD, dementia with Lewy bodies, exhibiting similar burden to controls. On the other hand, patients presenting dementia with DLB have shown higher amyloid burden than controls, comparable to levels seen in AD patients.^30^ ^,^ ^31^ Interestingly, these studies have suggested that a positive amyloid-PET scan in DLB individuals might be associated with more rapid clinical progression.^28^ ^,^ ^30^

The clinical importance of amyloid imaging has been extensively debated. In order to delineate scenarios in which the use of amyloid PET radiotracers is appropriate in the evaluation of cognitive impairment, the Alzheimer's Association and the Society of Nuclear Medicine and Molecular Imaging have set up an Amyloid Imaging Taskforce (AIT).^32^ Performing a literature review in conjunction with expert opinion, the AIT defined a set of Appropriate Use Criteria (AUC) for clinical amyloid imaging, recommending that the use of amyloid imaging be limited to patients showing progressive and unexplained cognitive decline with uncertain diagnosis, early onset dementia and/or atypical clinical presentation, and when knowledge of amyloid status is expected to alter the therapeutic approach. Clinical utilities of amyloid imaging are restricted to specific cases. Patients with early-onset dementia (commonly defined as onset before 65 years of age) have a lower probability of AD pathophysiology underlying their cognitive decline than late-onset cases. In this regard, amyloid imaging might clarify whether underlying brain amyloidosis is associated with clinical syndromes such as primary progressive aphasia, dementias characterized by a predominance of executive dysfunction, visuospatial symptoms, progressive apraxia, and corticobasal syndrome. Given the economic and family impact of the diagnosis of AD in this population, an elevated level of diagnostic certainty is highly desirable. Furthermore, the presence of amyloid pathology might provide the rationale for proposing the clinical use of cholinesterase inhibitors in this population. The AIT contraindicates the use of amyloid imaging in asymptomatic people or in those with a cognitive complaint but no clinical confirmation of impairment, for determining the severity of dementia and for non-medical reasons, such as insurance, legal or employment decisions.

Finally, recently incorporated into AD research diagnostic criteria, amyloid PET plays a major role in defining AD. The IWG criteria classified amyloid PET as a useful biomarker which supports clinical diagnosis, especially when the presentation is atypical.^33^ Hence, amyloid PET has increasingly been used in clinical trials, particularly for enriching study populations comprising individuals with a high probability of presenting AD or for monitoring target engagement of anti-amyloid therapies. However, the clinical utility of amyloid PET has been increasingly discussed, particularly in the absence of effective disease modifying agents.^32^ ^,^ ^34^ ^,^ ^35^ The ensuing paragraphs summarize progress regarding amyloid imaging in AD. 

**Figure 1:**
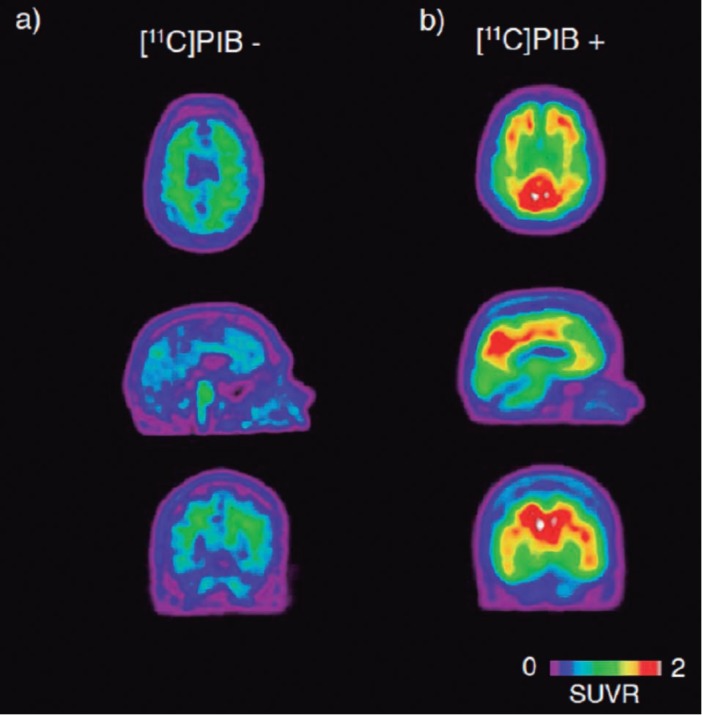
Amyloid signature. Representative [^11^C]PIB PET images showing white matter uptake of [^11^C]PIB in a patient with CBS (a; age 74, MMSE 23), and extensive cortical uptake in a patient with AD (b; age 70, MMSE 28).

[^11^C]Pittsburgh Compound-B. [^11^C]PIB exhibits a high affinity and specificity for Ab plaques, as opposed to Lewy bodies or tau proteins.^36^ ^,^ ^37^ showing high accuracy in the evaluation of Ab plaque burden. In individuals with AD, [^11^C]PIB uptake is distributed in the frontal, medial and lateral posterior parietal cortices, precuneus, occipital cortex and lateral temporal cortices, as well as in the striatum (Figure 1).^16^ ^,^ ^38^ ^-^ ^40^ Low [^11^C]PIB uptake is typically observed in the cerebellar cortex. 

A recent Cochrane review study on [^11^C]PIB for early diagnosis of AD in individuals with MCI^41^ estimated sensitivity of 96% (95% confidence interval: 87-99) and median specificity of 58%. A growing consensus emerging from longitudinal studies indicates that disease-modifying therapies targeting amyloid should be administered at very early stages of the disease.^42^ Concerted efforts worldwide are focusing on advancing the diagnosis of AD to a preclinical stage.^43^ ^,^ ^44^ Elevated [^11^C]PIB binding in nondemented subjects indicates that it may be sensitive for the detection of preclinical AD,^45^ suggesting a 20 to 30-year interval between first amyloid positivity and onset of dementia.^25^

Presently, [^11^C]PIB is one of the most accurate agents for localizing and quantifying Ab deposition; however, the short half-life of carbon-11 restricts its use to facilities with an onsite cyclotron and expertise in carbon-11 radiochemistry. Hence, the overall production and utilization costs are not generally affordable. In order to solve this issue, recent studies haves focused on fluorine-18 labeled radiopharmaceuticals, whose longer half-life allows for regional distribution and commercialization. Widely used as a biomarker in the diagnostic criteria for AD, cerebrospinal fluid (CSF) also confirms Ab pathology, with CSF Ab_1-42_ having an inverse correlation with [^11^C]PIB SUVR values.^46^ However, one limitation of CSF Ab_1-42_ is that it does not provide any regionalized information on amyloid burden in the brain.

Fluorine-18 labeled ligands. While the short half-life of carbon labeled radiotracers limits the application of [^11^C]PIB in clinical practice, fluorine-18 labeled amyloid PET radiopharmaceuticals have been developed, with a growing potential for clinical and research purposes. These compounds provide a new window of opportunity in the assessment of preclinical and clinical AD due to the 110-minute half-life of fluorine-18, a massive difference in terms of distribution logistics when compared to [^11^C]PIB production. At present, four radiofluorinated labeled radiopharmaceuticals are drawing scientific attention: [^18^F]3-F-PIB ([^18^F]flutemetamol),^47^ [^18^F]AV- 45 ([^18^F]florbetapir),^48^ [^18^F]AV-1 or [^18^F]BAY94-9172 ([^18^F]florbetaben),^49^ ^,^ ^50^ and [^18^F]AZD4694 or [^18^F]NAV4694.^51^

Similarly to [^11^C]PIB, these fluorine-18 amyloid tracers exhibit significant binding to fibrillar Ab in the brain,^47^ ^,^ ^48^ albeit with some differences. [^18^F]Flutemetamol, [^18^F]florbetapir, and [^18^F]florbetaben are less specific than [^11^C]PIB, displaying white matter binding whereas [^18^F]NAV4694 shows more specific grey-white matter demarcation and better pharmacokinetic properties as a potential competitor of [^11^C]PIB PET. The Food and Drug Administration (FDA) and European Medicines Agency (EMA) have already approved [^18^F]florbetapir (Amyvid^TM)^, [^18^F]flutemetamol (Vizamyl^TM)^, and [^18^F]florbetaben (Neuraceq(tm)) for use in clinical practice, while [^18^F]NAV4694 is currently in phase III trials.^50^ ^,^ ^51^

While holding great research potential, all four compounds discriminate healthy controls from AD subjects with high accuracy. [^18^F]Florbetapir has shown remarkable accuracy in amyloid detection, with clinical application even in preclinical AD.^52^ ^,^ ^53^ The diagnostic value of [^18^F]flutemetamol has been tested recently in symptomatic and preclinical AD, showing relevance in detecting primarily advanced stages of Ab deposition at both clinical and preclinical stages.^54^ [^18^F]Florbetaben is a highly accurate Ab PET tracer that has the potential to support the clinical diagnosis of AD and other causes of cognitive decline,^49^ with similar visual and quantitative assessment in PET.

A third generation probe, [^18^F]AZD4694, called NAV4694, has been attracting growing attention for its near-identical imaging characteristics , but longer half-life to those of [^11^C]PIB. [^18^F]NAV4694 binds specifically to Ab plaques, with excellent frontal cortex-to-white matter ratios both in AD and healthy controls.^51^ The accuracy of [^18^F]NAV4694 in binding Ab plaques has been tested, reliably discriminating AD patients from healthy controls, and satisfies requirements for clinical usage and evaluation of disease-modifying strategies in AD.^55^ In a recent study, [^18^F]NAV4694 exhibited a significant overlap in amyloid imaging with [^11^C]PIB in patients with FTLD, theoretically an Ab-free disease in most of its pathological subtypes.^56^ Also, the linear correlation of [^18^F]NAV4694 with [^11^C]PIB of 0.95 is higher than the values reported for [^18^F]florbetapir (range 0.33-0.64) and [^18^F]florbetaben (0.71).^51^

## PET BIOMARKERS FOR NEUROINFLAMMATION 

A factor known to be involved in the pathogenesis of AD is the immune response, with initial association between amyloid deposits and immune response emerging among elderly in their 70s and 80s.^57^ ^,^ ^58^ More recently, there is emerging knowledge on components of innate immunity associated with AD pathology, as well as increasing discussion over the beneficial and detrimental effects of the immune response in AD. Another controversial topic is the start point of neuroinflammatory processes observed in AD brains, which raises the question as to whether inflammation is a cause or a consequence of AD pathology. Despite the initial assumption of their occurrence only in late stages of the disease, inflammatory changes in the CSF can be detected in MCI patients, revealing the possibility of involvement of the immune system at very early stages of AD.^59^

In a bid to answer the as yet unsolved questions regarding the *Janus face* of inflammation in AD, PET imaging is being used *in vivo* to trace markers of neuroinflammation with promising outcomes, as outlined below.^60^

Radiopharmaceuticals for imaging microglial activation. Neuroinflammatory changes are a part of AD pathology, and microglial activation in areas affected by neurodegeneration is a key brain tissue event.^61^ Microglial activation occurring in early stages of AD dementia is associated with a significant elevation in the fractional area of reactive microglia following the formation of neuritic plaques comprising fibrillar Ab.^62^ ^-^ ^65^ Using a specific ligand for the 18-kDa translocator protein (TSPO), formerly called the peripheral benzodiazepine receptor (PBR), [^11^C]PK11195 quantification of microglial activation *in vivo* has been measured.^66^ Probably due to its poor specific binding, initial [^11^C]PK11195 studies in AD showed negative results.^67^ However, improvements in the [^11^C]PK11195 tracer, particularly with the utilization of its dextro-isomer, the radiotracer [^11^C]-(R)-PK11195, revealed increased sensitivity for detecting TSPO expression in parieto-temporal, entorhinal, and cingulate cortices of AD patients.^64^ In [^11^C]PIB+ AD patients, high microglial activation has been observed, with an inverse correlation between cognition and microglial activity.^68^ However, a recent study reported that the inclusion of a vascular component results in an amplified signal in AD patients,^69^ suggesting that an increase in [^11^C]-(R)-PK11195 sensitivity signal modeling may be required.

Other TSPO radiopharmaceuticals have been designed aimed at improving pharmacokinetics and specificity. In this regard, preclinical studies involving radiotracers [^18^F]FEDAA1106 or [^11^C]AC5216 have shown promising results in AD-like transgenic models,^70^ ^,^ ^71^ and [^11^C]DAA1106 PET imaging showed greater binding in AD patients compared to healthy control subjects.^72^ Another recent clinical study using [^18^F]FEDAA1106 - a novel TSPO ligand with *in vitro* affinity superior to that of [^11^C]DAA1106^73^ - showed widespread increases in MCI patients when compared to healthy controls, with these values predicting the conversion to AD dementia stage within a 5-year follow-up period.^74^

Although applications in studies of AD patients have not been performed, [^11^C]AC5216 has shown promising pharmacokinetic features and a higher affinity than [^11^C]PK11195 in healthy subjects.^75^ However, the potential clinical applications of TSPO radiotracers are limited by the rs6971 polymorphism in the TSPO gene, which confers lower uptake in polymorphism carriers (~30%) in comparison to non-carriers.^76^ Importantly, the cannabinoid receptor type 2 (CB2) was identified as a marker of microglial activation,^77^ leading to greater attention to the endocannabinoid system. In this respect, preclinical studies of CB2 using [^11^C]A-836339 showed upregulation in a transgenic model displaying cerebral amyloidosis.^78^

Radiopharmaceuticals for imaging reactive astrocytosis. In AD post-mortem tissue, the augmented expression of glial fibrillary acidic (GFAP) and astroglial S100B proteins is typically observed, indicating an increase in the number of reactive astrocytes.^79^ Utilizing a ligand with a high affinity/specificity for monoamine oxidase B (MAO-B), an enzyme expressed primarily on the mitochondrial membrane of reactive astrocytes,^80^ ^,^ ^81^ PET imaging using the carbon-11 labeled L-deprenyl ([^11^C]DED) has revealed increased binding in patients with MCI, suggesting that astrocytosis is an early event in AD pathophysiology.^82^

## PET BIOMARKERS OF NEURODEGENERATION

PET radiopharmaceuticals of tau pathology. Misfolding and aggregation of hyperphosphorylated tau deposition into NFTs has a key role in AD pathophysiology.^83^ ^,^ ^84^ It is has been proposed that tau pathology propagates across brain circuits. NFTs have been associated with neuronal dysfunction, cell death, and cognitive impairment.^85^ CSF levels of total tau (t-tau) and phosphorylated tau (p-tau) have been associated with disease severity,^86^ with altered tau levels in the CSF being interpreted as surrogate markers of neurodegeneration in AD.^87^ Despite this evidence, the clinical application of CSF tau as an AD biomarker has been further discussed elsewhere.^88^

CSF tau biomarkers are somewhat disadvantageous compared to imaging biomarkers given the need for a lumbar puncture. Moreover, CSF measurements provide global estimates of the disease process without any information regarding the topographic localization of NFT. Finally, the quantification of p-tau and t-tau protein varies significantly across centres.^89^ Due to these features, imaging methods for NFT quantification are extremely important, particularly for assessing upcoming tau-based therapeutics. Given this scenario, several radiotracers characterized by high affinity for tau fibrils and with suitable kinetics have been developed, including the benzothiazole derivative [^11^C]PBB3, the phenylquinoline derivatives-[^18^F]THK-523, [^18^F]THK-5105, and [^18^F]THK-5117-and benzimidazole pyrimidine derivatives, such as [^18^F]T807 and [^18^F]T808.^90^

[^11^C]PBB3 has shown to rapidly cross both the blood-brain barrier (BBB) and neuronal plasma membranes, binding to intraneuronal tau inclusions. *In vitro* and *ex vivo* autoradiographic studies have shown that [^11^C]PBB3 produced specific, high-contrast labeling of neuronal tau inclusions in the brain stem of mice models expressing human tau pathological mutations. The same findings were reported with *in vitro* autoradiography and AD tissue, showing evident radiolabelling of fibrillar aggregates in specific regions of the hippocampus (including CA1), and the frontal cortex. Similarly, a clinical PET study using [^11^C]PBB3 in probable AD patients revealed increased tracer binding in lateral temporal and frontal cortices, in line with the distribution of tau pathology at Braak stage V/VI, and with higher SUVRs correlating with lower memory scores. A slight increase in [^11^C]PBB3 retention was also observed around the hippocampus of a control subject who showed decline on the Mini-Mental State Examination (MMSE), consistent with Braak stage III/IV or earlier.^91^

Aside from [^11^C]PBB3, the fluorinated probes [^18^F]T-807 and [^18^F]T-808 are also potential tau ligands. An autoradiography study using AD brain tissue has shown that [^18^F]T-807 exhibits strong binding to NFTs, with a selectivity estimate of 29-fold for tau relative to Ab.^92^ In addition, comparing double immunohistochemical staining of NFTs and Ab on adjacent tissue sections, [^18^F]T-807 autoradiography showed that tracer binding co-localized with immunoreactive NFTs, not with Ab plaques.^93^ In the case of [^18^F]T-808, it also has a high affinity and good selectivity for NFTs over Ab, which was indexed by autoradiographic studies.^94^ In addition, [^18^F]T-808 presents fast brain uptake followed by a rapid washout, suggesting low non-specific binding,^94^ which is consistent with *in vivo* findings obtained using [^18^F]T-807.^93^ Moreover, the first human brain using [^18^F]T-807 showed elevated SUVR in AD compared to MCI and healthy control subjects. Distinct patterns of tracer accumulation, in line with Braak staging, was observed across the frontal, temporal and parietal cortices, as well as in the hippocampus/entorhinal region.^92^

Another class of fluorinated probes includes the THKs, which have exhibited high affinity and selectivity for tau aggregates.^95^ *In vitro* preliminary studies have reported [^18^F]THK-523 binding affinity for tau fibrils, with subsequent autoradiographic analysis using AD medial temporal brain sections showing accumulation of [^18^F]THK-523 in the pre- and pri-a layers of the entorhinal cortex and hippocampal CA1 region. Immunohistochemistry confirmed that these findings were consistent with the density of PHF-tau deposition.^96^ Subsequently, histofluorescence and autoradiographic studies revealed that [^18^F]THK-523 binding to NFTs co-localized with tau immunoreactivity, with no visible binding to Ab plaques.^97^ However, recent immunohistochemical and histofluorescence studies questioned [^18^F]THK-523's future in both research and clinical settings due to very high non-specific white matter binding.^98^ Moreover, preliminary clinical data have suggested that [^18^F]THK-523 does not bind tau inclusions in non-AD tauopathies.^99^

The second generation of THKs was developed, which includes [^18^F]THK-5105 and [^18^F]THK-5117. Binding studies conducted *in vitro* using [^18^F]THK-5105 and [^18^F]THK-5117 have found increased binding affinity to synthetic truncated tau (K18ΔK280) fibrils-comprising the four repeat regions (244-372) in the absence of lysine 280 (ΔK280)-with both tracers proving superior to [^18^F]THK-523. The selective binding capacity of these compounds was further examined using *in vitro* autoradiography and AD mesial temporal brain sections, showing increased accumulation of the radiotracer with particularly high binding in the Sommer's sector of the hippocampus, the parahippocampus, and the subiculum. These findings were confirmed through staining and immunohistochemistry, with reduced binding among healthy controls.^100^ Additional assessment of [^18^F]THK-5105, conducted using [^11^C]PIB and AD hemibrain sections, observed dense accumulation of [^18^F]THK-5105 in tau-rich areas-including the hippocampus/parahippocampus, insula, cingulate gyrus and inferior and middle temporal gyri-with the pattern of tracer binding corresponding to the recognized distribution of tau pathology but not to that of Ab or areas showing elevated retention of [^11^C]PIB. In addition, further studies of biodistribution conducted in normal mice showed profuse and rapid brain uptake and fast clearance, with the kinetics of both tracers superior to those reported for [^18^F]THK-523.^100^ ^-^ ^102^

**Figure 2:**
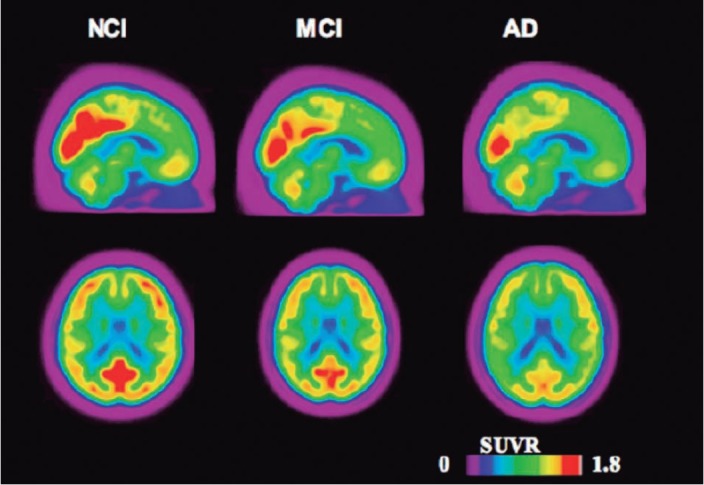
Representative PET [^18^F]FDG images acquired from a total of 103 individuals with normal cognition (NCI, N= 17), mild cognitive impairment (MCI, N=52) and dementia due to AD (AD, N= 27) obtained from a total of 103 structural MRI and [^18^F]FDG scans from the Alzheimer's Disease Neuroimage Initiative (ADNI) database. The hot color scale represents the magnitude of [^18^F]FDG standardized uptake value ratio (SUVR), proportional to glucose uptake. Note lower [^18^F]FDG SUVRs in MCI and AD as compared to controls. High SUVRs are particularly reduced in the posterior cingulate, precuneus and prefrontal (medial and dorsolateral) cortices. Imaging Methods: MRI images were corrected for non-linearity, classified (white, grey matter and CSF) and automatically segmented into cortical regions. Standard uptake value ratio (SUVR) images were calculated in PET native space using the cerebellum as a reference image. Images were subsequently resampled into the standard stereotaxic space and averaged using minctools.

[^18^F]FDG Radiotracer. The glucose analogue 2-deoxy-2-(^18^F)fluoro-D-glucose ([^18^F]FDG) PET has been utilized to assess cerebral glucose metabolism. [^18^F]FDG images have been interpreted as markers of neuronal activity^103^ and synaptic density.^104^ The typical AD metabolic signature consists of hypometabolism in the parieto-temporal association, medial temporal, posterior cingulate and frontal cortices, with relative preservation of the visual cortex, primary sensory motor cortices, basal ganglia, thalamus, and cerebellum (Figure 2).^105^ ^-^ ^109^ This hypometabolic signature is frequently observed in AD patients and in over 85% of pathologically-confirmed cases.^106^ However, in atypical focal cortical syndromes of AD, topographic variants of hypometabolism have been identified. For example, compared to typical AD, patients with logopenic primary progressive aphasia presented with disproportionate left temporoparietal hypometabolism.^110^ Patients with posterior cortical atrophy showed hypometabolism predominantly in occipito-parietal regions; some patients can present hypometabolism in the frontal eye fields.^111^ Patients with early onset AD also show greater metabolic reductions, with hypometabolism from mild dementia comparable to that observed in late onset cases with severe dementia.^112^ These findings are supported by studies showing more aggressive progression from patients with early onset AD^113^ and, possibly, by the cognitive reserve theory.^114^

In amnestic MCI (aMCI) patients, the pattern of hypometabolic changes usually occurs in brain regions classically affected in AD,^115^ but to a lesser degree.^116^ ^-^ ^118^ The patterns of brain metabolism in aMCI and non-amnestic MCI (naMCI) subjects are similar, however, aMCI has shown a decrease in medial temporal lobe metabolism and naMCI hypometabolism in the right prefrontal region.^119^ ^,^ ^120^ Some authors report that the anterior hippocampal formation can contribute to differentiating MCI patients from healthy control subjects, although other data refutes this, with the partial volume effect in the metabolism of the hippocampal formation on [^18^F]FDG imaging constituting a complicating factor.^121^ ^-^ ^125^ The posterior cingulate is the most relevant area for predicting conversion from MCI to AD, given that hippocampal hypometabolism is highly influenced by the atrophy observed on MRI and, after correcting for the partial volume effect, this finding is no longer supported.^117^ [^18^F]FDG has limited clinical value in MCI patients due to the lack of specificity for AD pathophysiology. At a population level, however, subjects with MCI presenting a more marked or 'AD-like' pattern have been found to convert to dementia at higher rates,^126^ ^,^ ^127^ with accuracies in the range of 75 to 100%.^128^ ^,^ ^129^ The magnitude of hypometabolism in the parietal and posterior cingulate cortices in MCI is associated with memory decline.^130^ ^,^ ^131^ As compared to decliners, stable MCI populations tend to exhibit hypometabolism restricted to the dorsolateral frontal cortex.^132^ ^,^ ^133^

In order to study the progression to MCI and AD among cognitively normal older individuals, [^18^F]FDG-PET has been used to predict cognitive decline with an accuracy approaching 80%.^121^ ^,^ ^123^ Progressive reductions in PET glucose metabolism were observed years before the appearance of clinical symptoms, with reductions in the hippocampus preceding declines in cortical regions^134^ in a clinicopathological study that evaluated cognitively normal individuals followed through MCI to pathologically-confirmed AD. The same metabolic changes have been noted in cognitively normal subjects homozygous for a susceptibility gene, the apolipoprotein E (APOE) e4 allele,^130^ ^,^ ^135^ asymptomatic carriers of genetic mutations associated with early onset familial AD,^128^ ^,^ ^129^ ^,^ ^136^ and those with a maternal family history of AD, as compared to those with a paternal history or no family history of AD.^137^ Ultimately, [^18^F]FDG will potentially prove of use in the characterization of a subgroup of patients exhibiting neurodegeneration in the absence of Ab deposition.^138^ ^,^ ^139^ These patients, classified in the category of "suspected non-amyloid pathophysiology (SNAP)", could suggest that the onset of neurodegeneration in AD may not depend on the accumulation of Ab.^140^

## IMAGING ALZHEIMER'S DISEASE PATHOPHYSIOLOGY IN EXPERIMENTAL MODELS

A miniaturized version of PET, termed microPET, has made non-invasive imaging of small animals possible, such as rats and mice. In addition, advances in genetic engineering have led to the development of diverse animal models harboring human pathological gene mutations, which are capable of mimicking amyloid and tau pathologies (for review see (141)). These models show progressive deposition of amyloid or tau, in parallel with significant cognitive decline, and are highly suited to longitudinal assessment with microPET. 

To date, several studies have investigated AD pathophysiological events in rodent models with microPET (for review see (142)). However, few such studies have been conducted using a longitudinal design. Recently, the first prolonged longitudinal study with microPET evaluating amyloid was published and revealed non-linear patterns of amyloid deposition during full disease progression.^143^ By contrast, to our knowledge, there are no longitudinal studies in the literature following tau pathology, and such studies are anxiously awaited by the AD community.^144^ In this context, longitudinal studies associating these animal models and microPET imaging have high translational capability, which indicates that data collected can be rapidly translated to clinical studies. In addition, microPET longitudinal studies offer unprecedented opportunities for monitoring the effectiveness of innovative therapeutic strategies. 

## CONCLUSION

Predictions based on biomarkers indicate that AD pathophysiological abnormalities precede the onset of clinical symptoms by at least two decades. By obtaining earlier AD diagnosis, it will be possible to develop potential innovative therapies that may impact the natural progression of AD. In fact, several clinical studies testing potential new drugs are currently underway with amyloid and tau being the most promising pharmacological targets (for review see (145)). In this scenario, PET radiotracers and neuropathological features for these processes are crucial to determine amyloid and tau engagement and to assess treatment response in clinical trials. In keeping with this, radiopharmaceuticals for neuroinflammatory molecular imaging can equally contribute to the development of potentially effective anti-inflammatory interventions. Although the majority of PET studies in AD populations have focused on Ab imaging, several 'non-amyloid' radiopharmaceuticals exist for evaluating neurodegeneration, neuroinflammation and perturbations in neurotransmission across the spectrum of AD, potentially contributing to improved therapeutic perspectives for AD (for review see (146)).

At present, neurology is experiencing a new era which encompasses imaging assessment for patients suspected of having AD. Thus, it is extremely important to accurately establish those patients who are candidates for performing a cerebral PET exam (e.g. amyloid imaging). In the case of amyloid positivity, this biomarker-based information reflects an elevated risk for AD, and health professionals should exercise caution when ordering this test in non-demented patients (e.g. healthy subjects and MCI patients). In the coming few years, when more effective therapies are likely to become available, amyloid imaging will be increasingly applied to identify patients with early AD or at risk of developing AD; preferably in association with [^18^F]FDG imaging in order to combine pathological (amyloid deposition) and metabolic (hypometabolism) information. Other radiotracers will also play a key role for research in AD (Table 2), especially in targeting novel therapies and for monitoring the response and efficacy of new drugs (e.g. tau and neuroinflammation). 

**Table 2:** PET imaging signatures in Alzheimer's disease.

Biological target	Radiotracers	Findings	Typical brain regions involved
Amyloid deposition	[^11^C]PIB [^18^F]Florbetapir [^18^F] Florbetaben [^18^F] Flutemetamol [^18^F]NAV4694	Increased retention	Frontal cortex, medial and lateral posterior parietal cortices, precuneus Occipital cortex, lateral, temporal cortices and striatum
Tau pathology	[^18^F]T807 [^18^F]T808 [^18^F]THK523 [^18^F]THK5105 [^18^F]THK5117 [^11^C]PBB3	Increased retention	Frontal cortex, temporal cortex, parietal cortex and hippocampus/entorhinal region
Glucose metabolism	[^18^F]FDG	Low uptake	Parietotemporal association cortices, medial temporal cortex, posterior cingulate and frontal cortex (at later stages)
Neuroinflammation	[^11^C]PK11195 [^11^C]DAA1106 [^18^F]FEDAA1106 [^11^C]AC5216 [^11^C]A836339 [^11^C]L-deprenyl	Increased retention	Widespread retention within the whole brain
